# Managing patients taking novel oral anticoagulants (NOAs) in dentistry: a discussion paper on clinical implications

**DOI:** 10.1186/s12903-016-0170-7

**Published:** 2016-01-28

**Authors:** Fulvia Costantinides, Roberto Rizzo, Lorenzo Pascazio, Michele Maglione

**Affiliations:** School of Specialization in Oral Surgery, Department of Medical, Surgical and Health Sciences, University of Trieste, Trieste, Italy; Unit of Geriatrics, Department of Medical, Surgical and Health Sciences, University of Trieste, Trieste, Italy

**Keywords:** Novel oral anticoagulants, Dabigatran, Rivaroxaban, Apixaban, Dental treatment

## Abstract

**Background:**

The aim of this paper is to contribute to the discussion on how to approach patients taking new orally administered anticoagulants (NOAs) dabigatran etexilate (a direct thrombin inhibitor), rivaroxaban and apixaban (factor Xa inhibitors), before, during and after dental treatment in light of the more recent knowledges.

**Discussion:**

In dentistry and oral surgery, the major concerns in treatment of patients taking direct thrombin inhibitors and factor Xa inhibitors is the risk of haemorrhage and the absence of a specific reversal agent. The degree of renal function, the complexity of the surgical procedure and the patient’s risk of bleeding due to other concomitant causes, are the most important factors to consider during surgical dental treatment of patients taking NOAs. For patients requiring simple dental extraction or minor oral surgery procedures, interruption of NOA is not generally necessary, while an higher control of bleeding and discontinuation of the drug (at least 24 h) should be requested before invasive surgical procedures, depending on renal functionality.

**Summary:**

The clinician has to consider that the number of patients taking NOAs is rapidly increasing. Since available data are not sufficient to establish an evidence-based dental management, the dentist must use caution and attention when treating patients taking dabigatran, rivaroxaban and apixaban.

## Background

In the last few years, new orally administered anticoagulants drugs (NOA) have been introduced in clinical practice for patients affected by various diseases and medical conditions that require use of extended-duration anticoagulant therapy (prophylaxis and treatment of pulmonary embolism and venous thrombosis, including prophylaxis after orthopaedic surgery; prophylaxis and treatment of thromboembolic complications associated with atrial fibrillation and/or prosthetic valves replacement; reduction of the risk of death, reinfarction and thromboembolic events after myocardial infarction) [1]. Similarly to subcutaneous or intravenously administered low-molecular-weight heparin (LMWH) and in contrast to coumarin derivatives (warfarin and acenocoumarol), these new medications interfere with very specific steps of the coagulation cascade. Three types of NOAs have recently been approved for use in the USA and in several European countries, including Italy. These are dabigatran etexilate, which acts as a direct thrombin inhibitor (DTI), rivaroxaban and apixaban that work as factor Xa inhibitors (FXaI). A fourth one FXaI, edoxaban, obtained the recent approval of the European Medicines Agency in Europe (April 2015, 23th) [2].

Advantages of dabigatran, rivaroxaban, apixaban and edoxaban have to be researched in their capacity to provide a stable anticoagulation at a fixed dose without the necessity to monitor the coagulation with routine laboratory exams (INR). They have a relatively rapid onset and reach peak concentration in few hours [3]. Furthermore, unlike vitamin K antagonists, they show a wide therapeutic margin, low drug- to- drug interactions and no significant food interactions [1, 4].

The progressive diffusion of NOAs has a direct repercussion on different dentistry specialties particularly in a surgical context. Because of their relatively recent introduction, specific studies regarding dental treatment of patients taking NOAs are available in literature only from 2012. No data exist regarding dental management of patients treated with edoxaban. The aim of this paper is to contribute to the discussion on how to approach patients taking dabigatran, rivaroxaban or apixaban, before, during and after dental treatment in light of the more recent knowledges. For this purpose, a comprehensive search of the literature carried out through PubMed (www.ncbi.nlm.nih.gov/pubmed) Pubmed Central (http://www.ncbi.nlm.nih.gov/pmc/), Medline (http://www.nlm.nih.gov/bsd/pmresources.html) and Cochrane databases (http://www.cochranelibrary.com/), was performed from inceptions to the last access in August 2015. “Dabigatran”, “Rivaroxaban”, “Apixaban”, “Anticoagulants”, “Dental”, “Oral”, “Surgery”, combined with the Boolean operator ‘AND’ were used as search algorithm. Studies that provided general and specific information on NOAs in a dental context have been identified and selected.

## Discussion

### Dabigatran etexilate (Pradaxa®)

Dabigatran etexilate is a specific, reversible DTI that, after oral administration, is rapidly absorbed and converted in its active form, dabigatran, through esterase-catalyzed hydrolysis in plasma. Mechanism of action of dabigatran is to bind with the active site on free and clot-bound thrombin (factor IIa) so it cannot transform fibrinogen into fibrin [5]. It has a rapid onset of action with a peak plasma concentration at 0.5–4 h. The half –life elimination is 12–14 h in healthy patients, 14–17 h in elderly and up to 27 h in patient with severe renal dysfunction (creatinine clearance <15–30 ml/min) [6]. Dabigatran is not a substrate of the hepatic cytochrome P-450. The efficacy of dabigatran has been firstly assessed by the RE-LY trial in 2009 [7]. Results showed that, on a total of 18113 patients affected by atrial fibrillation recruited for the study, a dabigatran dose of 150 mg twice daily was associated to lower rates of stroke and systemic embolism but similar rates of major bleeding when compared with warfarin assumption. At a 110 mg twice daily, dabigatran showed similar rates of stroke and systemic embolism when compare with warfarin but with significantly lower cases of major bleeding. The most important finding was that the rate of hemorrhagic stroke with both doses of dabigatran was less than one-third that of warfarin, without any reduction in the efficacy against ischemic stroke [7].

Although the prescription cost of dabigatran is significantly higher than that of warfarin, the overall cost to the health care system seems to be more cost-effective than warfarin [8].

Monitoring of dabigatran is generally unnecessary; however, in case of emergency, the thrombin clotting time (TT) and the ecarin clotting time (ECT) are the most sensitive tests for quantifying anticoagulation rate. The activated partial thromboplastin time (aPTT) is less sensitive, especially for higher doses of dabigatran but anyway recommended in case of emergency situation owing to its broad availability [9, 10]. The INR test is insensitive to the activity of this drug and might not be elevated in patients taking dabigatran [11].

For patients who experience minor bleeding events, the delaying of the next dose or the discontinuation should be indicated although this choice has to be evaluated with prudence considering the possible risk of ischemic events [12]. For moderate or severe bleeding, treatments include mechanical compression, surgical intervention, fluid replacement, hemodynamic support, oral charcoal application (for recent ingestion of dabigatran) and haemodialysis. For life-threatening bleeding, treatment includes administration of prothrombin complex concentrates or charcoal and supportive measures [13] although recent investigations showed no effect of prothrombin complex concentrates on aPTT, TT and ECT [12]. In October, 2015, the U.S. Food and Drug Administration approved idarucizumab, a monoclonal antibody fragment (Praxbind®, Boehringer Ingelheim Pharmaceuticals, Inc., Germany) for the treatment of patients taking dabigatran etexilate when reversal of the anticoagulant effects of dabigatran is needed for emergency surgery/urgent procedures, or in life-threatening or uncontrolled bleeding [14]. Idarucizumab rapidly and completely reversed (within minutes) the anticoagulant activity of dabigatran in 88 to 98 % of patients in an initial clinical study [15]. The approval of idarucizumab will substantially influence the approach to haemorrhagic complications related to dabigatran probably solving the concerns that had been raised for the lack of a proven reversal strategy with respect to severe bleeding events in patients taking this NOA [12, 16].

Discontinuing anticoagulants, including dabigatran, for elective surgery or invasive procedures places patients at an increased risk of stroke and, if discontinuation is necessary, dabigatran should be restarted as soon as possible [17].

#### Perioperative bleeding associated to dabigatran etexilate

In the RE-LY trial (dabigatran versus warfarin in the treatment of atrial fibrillation), the rate of major bleeding associated with an invasive procedure or surgery was expressed as percentage [18]. Major bleeding was similar with dabigatran 110 mg twice daily (3.8 %), dabigatran 150 mg twice daily (5.1 %), and warfarin (4.6 %). Bleeding was greater in those undergoing urgent surgery (18 %, 18 %, and 22 %, respectively). The risk of major bleeding was increased in those who received bridging anticoagulation compared with those who did not, an effect that was seen both with dabigatran (major bleeding with and without bridging, 6.5 % versus 1.8 %) and warfarin (6.8 % versus 1.6 %) [19].

#### Perioperative thromboembolic risk associated to dabigatran etexilate

Of the 4591 patients who underwent elective procedures or surgery in the RE-LY trial, the perioperative thromboembolic risk was 1.2 %, based on a composite endpoint of stroke, cardiovascular death, and pulmonary embolism. There were no differences in thromboembolic risk with dabigatran versus warfarin, or with the high versus the low dabigatran dose. However, urgent surgery was associated with a higher risk of ischemic stroke or systemic embolism than elective surgery (warfarin: 1.8 % versus 0.4 %; dabigatran 150 mg twice daily: 1.4 % versus 0.4 %; dabigatran 110 mg twice daily: 2.8 % versus 0.3 %) [18].

### Dental consideration for patients taking dabigatran

The major concern in treatment of patients taking DTIs is the risk of haemorrhage in dentistry and oral surgery too. The recent approval of idarucizumab will help in solving the concerns related to the absence of a specific reversal agent for dabigatran. To date, there have been no published studies on the incidence of bleeding events among patients receiving dabigatran. However, several studies demonstrated that the risk of haemorrhage for patients taking DTIs is similar to those taking warfarin when the INR value ranges between 2 and 3 (except for gastrointestinal bleeding) [7, 18, 20]. For patients requiring simple dental extraction or minor oral surgery procedures (as localized surgical extraction, localized periodontal surgery, apicectomies, incisional biopsies or excision of localized mucosal lesion), it can be assumed that the risk is similar to that of patients taking vitamin k antagonists and with an INR < 3 [12]. One of the first rules to reduce the risk of bleeding, is to perform the surgical procedures as late as possible after the most recent assumption of dabigatran. After surgery, local haemostatic measures such as suturing, gelatine sponge or cellulose mesh, tranexamic acid mouth rinse (twice daily for 2–5 days) may help in controlling postoperative bleeding [21, 22]. Elective invasive surgical procedures as multiple surgical extractions, removal of extensive intraosseous lesions or maxillofacial surgery, should request an higher control of bleeding and even the decision of discontinuation of the drug because some dental patients may have a higher risk of bleeding when taking dabigatran [6]. A patient with high bleeding risk in a surgical context is a subject that could develop hemorrhagic complications (also life-threatening) that need important haemostatic interventions (like tissue or vascular sutures, use of pro-coagulants or reversal agents). Several factors concur in the assessment of bleeding risk and these are patient-dependent or surgery-dependent. Patient-dependent factors are age, renal functionality, congenital or acquired alterations of the coagulation, assumption of antiplatelet or anticoagulant drugs (i.e. patients older than 75 years and taking acetylsalicylic acid, long term NSAIDs or clopidogrel or prasugrel) [23]. Surgery-dependent factors are correlated to the invasiveness and difficulty of the surgery. To assess the risk of bleeding, patient-dependent factors and surgery-dependent factors must be considered together. In this sense an accurate anamnesis and surgical planning are extremely important to intercept these high- risk categories. Actually, the best available protocol regarding discontinuation of dabigatran in elective surgery is that proposed by van Ryn et al. [9]. It takes into account the degree of renal function, the complexity of the surgical procedure and the patient’s risk of bleeding due to other concomitant causes (Table 1). Additionally, TT or aPTT performed 6 to 12 h prior to surgical treatment can indicate, when normal, if the anticoagulant effect of dabigatran has resolved [6].

**Table 1:** Guide to discontinuation of dabigatran before elective surgery; indications are matched for renal function and risk of bleeding (van Ryn et al. 2010) [9]

Cretinine clearance (ml/min)	Time of discontinuation before surgery for standard risk of bleeding	Time of discontinuation before surgery for high risk of bleeding^a^
>80	24 h	2–4 days
>50 to ≤ 80	24 h	2–4 days
>30 to ≤ 50	≥48 h	4 days
≤30^b^	2–5 days	5 days

^a^Determinants of bleeding risk are: type of surgery (cardiac, neural, abdominal, surgery involving major organs or requiring complete haemostasis), advanced age, comorbidities (i.e. major cardiac, respiratory, liver diseases) and concomitant use of antiplatelet therapy

^b^Dabigatran controindicated

Dabigatran should be restarted once a stable clot has formed, usually within 24–48 h following surgery, because of the rapid onset of this drug [1].

Anyway, all the modifications of the therapy with dabigatran have to be discussed with the patient’s physician in terms of risks-benefit to optimize the surgical treatment without increasing the risk of thromboembolism. In patients needing a maintenance of anticoagulation during extensive major oral surgery, a switch to LMWH should be considered [12]. Furthermore, in the postoperative phase, attention has to be paid to the administration of analgesics. Although dabigatran does not directly interact with NSAIDs, both increase the risk of bleeding [12]. For this reason, prescription of NSAIDs should be made with caution, preferring alternative drugs for pain management. Paracetamol or opioid medications are safer alternatives for patients taking dabigatran [12].

Dabigatran acts as a substrate of P- glycoprotein 1 (P-gp 1), an important protein of the cell membrane that pumps many foreign substances out of cells. Concomitant assumption of strong P-gp 1 inducers like rifampicin, dexamethasone and carbamazepine, has been reported to significantly decrease the plasma concentration-versus time curve and peak serum concentration of dabigatran. For this reason, these drugs are not recommended in patients taking DTIs [1]. A single dose of 400 mg of ketoconazole, a P-gp 1 inhibitor, was reported to increase the peak plasma concentration of dabigatran to 135 % while concomitant therapy with clarithromycin, a moderate P-gp 1 inhibitor, has substantially no effects on dabigatran concentrations. The administration of P-gp 1 inhibitors like ketoconazole (and possibly itraconazole, erythromycin, clarithromycin) should be avoided or, if necessary, a reduced dose of dabigatran may be indicated [1].

### Rivaroxaban (Xarelto®)

Rivaroxaban is an orally administered selective, reversible direct FXaI that binds with the part of factor XaI that catalyzes the activation of prothrombin (factor II) [1]. Rivaroxaban can inhibit free factor Xa but also clot-bound factor Xa and factor Xa bound to the prothrombinase complex [5]. The drug shows a rapid onset and reaches the peak plasma concentration in 2.5–4 h after oral administration (once daily). The half-life is 5.7–9.2 h (up to 12–13 h in patients older than 75 years) and approximately 51 % of rivaroxaban undergoes metabolic degradation without involving cytochrome P-450. FXaIs, including rivaroxaban, slightly prolong prothrombin time (PT) and aPTT. No routine monitoring of rivaroxaban is generally required however, in case of an emergency, anti factor Xa is reported as the most accurate measurement of anticoagulation.

Rivaroxaban is indicated in the prevention of venous thromboembolism in adults subjected to elective hip or knee replacement surgery, in the treatment of deep venous thrombosis (DVT) and pulmonary embolism following DVT in adults patients [4]. The efficacy of rivaroxaban in comparison with warfarin, was tested in the ROCKET-AF study, a large clinical trial that assessed the effectiveness of the drugs in reduction of ischemic stroke or systemic embolism in patient affected by atrial fibrillation [24]. Recent meta-analysis and systematic reviews showed that FXaI significantly reduces the number of strokes and systemic embolic events compared with warfarin in patients with atrial fibrillation. FXaI also seems to reduce the number of major bleedings and intracranial haemorrhages compared with warfarin [25, 26].

Rivaroxaban has not a specific antidote [1] although some studies suggest the use of recombinant factor VIIa or active concentrate prothrombin complex to antagonizing the anticoagulant effect [27]. Recently, a modified form of factor Xa is being studied as a potential antidote for factor Xa inhibitors. This recombinant protein (r-Antidote, PRT064445, Portola Pharmaceuticals) is currently undergoing clinical trials for effectiveness of reversal of the effects of oral factor Xa inhibitors (rivaroxaban and apixaban) [28].

#### Perioperative bleeding associated to rivaroxaban

Minor bleeding events have been observed in 4–7 % of patients, while < 1–2 % experienced major bleeding events [29].

In the ROCKET AF trial (rivaroxaban versus warfarin for atrial fibrillation), the rate of major bleeding associated with an invasive procedure or surgery was 2.3 % [30]. Bleeding was similar between the two anticoagulants, and between patients who received bridging anticoagulation and those who did not.

#### Perioperative thromboembolic risk associated to rivaroxaban

In the ROCKET AF trial, a randomized, double-blind, double-dummy study of rivaroxaban and warfarin in non-valvular atrial fibrillation, baseline characteristics, management, and outcomes, including stroke, non-central nervous system systemic embolism, death, myocardial infarction, and bleeding, were reported in participants who experienced temporary interruption (TI, 3–30 days) for any reason [30]. The at-risk period for outcomes associated with TI was from TI start to 30 days after resumption of study drug. In 14236 participants who received at least 1 dose of study drug, 4692 (33 %) experienced TI. Participants with TI were similar to the overall ROCKET AF population in regard to baseline clinical characteristics. Only 6 % (*n* = 483) of TI incidences involved bridging therapy. Stroke/systemic embolism rates during the at-risk period were similar in rivaroxaban-treated and warfarin-treated participants (0.30 % versus 0.41 % per 30 days).

Rivaroxaban is the only NAO, with a study that considered interruption of treatment, for apixaban and dabigatran further studies are needed.

#### Dental consideration for patients taking rivaroxaban

To date, there are no clinical trials supporting specific measures in the event of haemorrhage in dental patients taking rivaroxaban. The most current informations suggest that interruption of rivaroxaban is not required for simple surgery like dental extraction [1, 31]. In general, the indications given for dabigatran are also applicable to rivaroxaban. Therefore, the drug can be normally administered without discontinuation, for uncomplicated extractions and other similar dental procedures in patients with normal renal functionality [6]. As dabigatran, rivaroxaban should be discontinued at least 24 h before oral and maxillofacial surgery in patients at risk of excessive bleeding or impaired haemostasis [1]. Patients with severe renal impairment may necessitate a period > 24 h of discontinuation because they show a significant increase in maximum plasma concentration and longer half- life of rivaroxaban [1]. When discontinued, rivaroxaban should not be restarted immediately after surgery, but usually within 24–48 h due to its rapid onset [1]. As for dabigatran, the decision of discontinuation has to be carefully discussed with the patients’s physician to avoid possible thrombo-embolic complications.

PT and APTT are not clinically useful in assessing the anticoagulant effect before oral surgery in patients taking rivaroxaban [1, 17].

When prescribing medical therapy for the treatment of oral and dental pathologies, clinician has to remember that two third of rivaroxaban is metabolized by cytochrome P-450 system (in particular by cytochrome P3A4) and it is also a substrate of P-gp transporters [6]. Concomitant use of strong CYP3A4 and P-gp inhibitors such as ketoconazole, itraconazole, voriconazol and posaconazole can increase serum concentration of rivaroxaban and should be avoided. Eventually, only fluconazole can be coadministered with caution [6]. Clarithromycin, a strong CYP3A4 inhibitor and a moderate P-gp inhibitor, slightly increases peak serum concentration of rivaroxaban when administered with a dose of 500 mg twice daily. Also erythromycin (a moderate CYP3A4 and P-gp inducer) does not lead to a clinically significant increase of serum concentration of rivaroxaban when taken at a dose of 500 mg, times per day [1]. Conversely, rifampicin, a CYP3A4 inducer, may increase the metabolism of rivaroxaban decreasing the level of anticoagulation [6].

There are no reports of interaction between rivaroxaban and amoxicillin, cephalexin, cefazolin, ampicillin or clindamycin [17]. Therefore, none of these antibiotics can be used safely.

No clinically relevant interaction has been observed between rivaroxaban and NSAIDs, although a study by Kubitza et al. [32] showed a significant increase in bleeding time for patients taking rivaroxaban and naproxen in comparison of those taking rivaroxaban alone. However, the increased bleeding time resulted not clinically relevant. It has also been observed that patients co-medicated with opioid drugs experienced higher major or non-major clinically relevant post-surgical bleeding compared with those co-medicated with NSAIDs. Based on these informations, prudence is needed when using NSAIDs and opioid drugs in patients taking rivaroxaban. A careful monitoring of the entity of bleeding is indicated in these patients especially during and after surgical dental procedure [1].

### Apixaban (Eliquis®)

Apixaban is the most recently introduced direct anticoagulant. It is a reversible orally administered FXaI with the same therapeutic indications of dabigatran and rivaroxaban. After intake, the peak plasma concentration is reached in about 1–3 h with an oral bioavailability of approximately 60 %. The half-life of the drug is about 12 h and it is excreted almost totally in bile [4]. As rivaroxaban, no specific reversal agent exists for apixaban. In case of emergency situations, recombinant factor VIIa, recombinant factor Xa or activated thrombin complexes can be used. In situations of normal bleeding, the delay or discontinuation of the next dose can be sufficient to resolve bleeding. However, few data are available for correct management of apixaban in case of haemorrhage and further studies are needed in this respect.

#### Perioperative Bleeding associated to apixaban

Data of ARISTOTLE trial (apixaban versus warfarin for atrial fibrillation) [33] showed that the rate of major bleeding associated with an invasive procedure or surgery was 1.8 %. Major bleeding was similar in those receiving apixaban or warfarin (1.6 % versus 1.9 %), and in those who continued or interrupted apixaban (1.6 % versus 1.7 %). In the warfarin arm, the rate of major bleeding was 3.0 % in those who continued warfarin versus 1.3 % in those who interrupted it. Many of the procedures were low risk (dental extraction, ophthalmologic surgery, colonoscopy or upper endoscopy).

#### Perioperative thromboembolic risk associated to apixaban

During 9260 procedures performed on patients in the ARISTOTLE trial, the perioperative thromboembolic risk was 0.57 % for warfarin and 0.35 % for apixaban [33].

### Dental considerations for patients taking apixaban

Because apixaban is one of the most recently marketed direct anticoagulant (2013), few data or guidelines are available for management of dental patient taking this drug. In general, the same indications used for dabigratran and rivaroxaban can be valid. Actually, there is no necessity to suspend apixaban before dental surgery in patients with no comorbidities for increased bleeding but further studies are needed in this respect. Similarly to rivaroxaban, administration of potent CYP3A4 and P-gp inhibitors are contraindicated [4].

### Final considerations

Summary of main characteristics of dabigratran etexilate, rivaroxaban and apixaban is shown in Table 2. Table 3 summarizes the more recent guidelines for dental management of patients taking NOAs.

**Table 2:** Principal characteristics of new oral anticoagulants (NOAs)

NOAs	Class	Indications	Dosage	Time to peak plasma concentration	Half life	Routes of elimination	Monitoring of coagulation
Dabigatran etexilate	Direct thrombin inhibitor	Prevention of cerebrovascular complications in non-valvular atrial fibrillation; hip and knee replacement surgery; venous thromboembolism prophylaxis and management	110 mg- 150 mg twice daily	2–4 h	12–14 h; 14–17 h in elderly; 15–18 h in moderate renal impairment; up to 28 h in advanced renal impairment	80 % renal, 20 % hepatic	Not needed
Rivaroxaban	Direct inhibitor of factor Xa	Prevention of cerebrovascular complications in non-valvular atrial fibrillation; venous thromboembolism prophylaxis and management	20 mg daily	2.5–4 h	5–10 h; 12–13 h in patients > 75 years	66 % renal, 28 % in feces	Not needed
Apixaban	Direct inhibitor of factor Xa	Prevention of cerebrovascular complications in non-valvular atrial fibrillation; venous thromboembolism prophylaxis and management	5 mg twice daily	1–3 h	10–14 h	25 % renal, 55 % intestinal, remnant hepatic	Not needed

**Table 3:** Summary of the more recent guidelines for dental management of patients taking NOAs

Author	Type of NOA	Minor surgical procedures (low-medium risk)^a^	Major surgical procedures and/or co-morbidities (high risk)^b^
Firriolo FJ and Hupp WS, 2012 [1]	Dabigatran	For dental procedure that involve bleeding: do not discontinue the daily dose in patient with normal renal function and without other risk for impaired haemostasis	For oral and maxillofacial surgical procedures with possible complications for excessive bleeding and/or impaired haemostasis: discontinue dabigatran ≥ 24 h before surgery or longer depending on renal impairment and bleeding risk (Table 1). Restart the drug at least 24 h postoperatively.
	Rivaroxaban	For dental procedure that involve bleeding: do not discontinue the daily dose in patient with normal renal function and without other risk for impaired haemostasis	For oral and maxillofacial surgical procedures with possible complications for excessive bleeding and/or impaired haemostasis: discontinue rivaroxaban ≥ 24 h before surgery or longer depending on renal impairment and bleeding risk (Table 1). Restart the drug at least 24 h postoperatively.
Davis C et al., 2013 [12]	Dabigatran	Perform surgery as long as possible after last dose Use local haemostatic measures	Discontinue 2–3 half –lives before surgery Adjust time of discontinuation for renal impairment
Hong CH and Islam I, 2013 [36]	Dabigatran, Rivaroxaban, Apixaban	Do not change administration Use local haemostatic measures	Suspend administration 24 h before surgery and restart drugs after complete haemostasis is achieved at least after 24 h post-operatively
Breik O et al., 2013 [37]	Dabigatran	Do not discontinue the drug Use local haemostatic measures (mechanical pressure, suturing an local haemostats)	In consultation with the patient’s physician, consider discontinuing the drug 24 h before procedure (or ≥ 48 h depending on degree of renal impairment) or changing to another anticoagulant preoperatively. Consider checking aPTT preoperatively Restart dabigatran 24–48 h after operation.

^a^Low-medium risk: local anaesthetic infiltration; simple single extraction; soft tissue biopsy ≤ 1 cm; supragingival prophylaxis; placement of rubber dam; restorations; crown preparation; root canal therapy; prosthetic rehabilitation of implant; band and brackets removal; wire insertion. Medium risk: local anaesthesia nerve block; multiple simple extractions ≤ 5 teeth; soft tissue biopsy 1–2.5 cm; placement of single implant; ultrasonic scaling; one to two quadrants (6–12 teeth) deep subgingival scaling; localize periodontal surgery ≤ 5 teeth (Hong and Islam, 2013) [36]

^b^High risk: multiple extraction > 5 teeth; surgical extraction requiring periosteal flap and ostectomy; soft tissue biopsy > 2.5 cm; osseous biopsy; removal of torus; placement of multiple implants; full mouth disinfection with deep subgingival cleaning; periodontal surgery > 5 teeth; endodontic surgery with osseous manipulation (Hong and Islam, 2013) [36]. Co-morbidities: presence of renal impairment; advancing age; major cardiac, respiratory or liver diseases; concomitant use of antiplatelet therapy (van Ryn et al., 2010) [9]

The analysis of the literature showed that the interindividual pharmacokinetic variability of NOAs is large. The four drugs that are most advanced in their clinical development and regulatory proceedings (dabigatran etexilate, rivaroxaban, apixaban and edoxaban) differ in their rate and extent of digestive adsorption and in the mechanisms and rate of elimination, predominantly renal or hepatobiliary.

However, the absorption, distribution, metabolism and elimination of these drugs are governed by many variables: liver and/or renal function, sex, weight, age, genetic polymorphisms of enzyme or efflux systems, drug-drug interference, etc. It follows that for a given patient, the residual concentration at a given time after halting treatment cannot be accurately calculated from the average values of the pharmacokinetics in the target population.

Once the thromboembolic and bleeding risks have been estimated, a decision can be made about whether the anticoagulant should be interrupted or continued. Data comparing the relative benefits of continuing anticoagulation versus interrupting an anticoagulant are limited, and decisions that balance thromboembolic and bleeding risks must be made on a case-by-case basis. No scoring system can substitute for clinical judgment in this decision-making.

Dental procedures are generally considered to confer a low risk of bleeding, and anticoagulation can be continued in most patients during these procedures. The evidence of continuing anticoagulation during selected low bleeding risk surgery comes from patients receiving warfarin with an INR in the therapeutic range [34, 35]. Conversely, the anticoagulant should be discontinued if the surgical bleeding risk is considered high (i.e. multiple extractions >5, surgery lasting more than 45 min, head and neck cancer, extensive oral and maxillofacial surgery in patients with comorbidities) [4, 36]. If a concomitant very high or high thromboembolic risk exists, the period without anticoagulation should be limited to the shortest possible interval. Unlike patients taking vitamin K antagonist, bridging with LMWH could be not required for those with very high or high thromboembolic risk who are receiving a DTI or FXaIs, in light of the shorter half-lives of these agents and the recent introduction of DTI reversal agent [12, 14].

NOAs are seeing increased use in Europe and USA, thanks to their efficacy and benefits. However, few data are still available regarding dental management of patients taking dabigatran, rivaroxaban, apixaban and edoxaban. The lack of data with regards to bleeding risk in dental treatment is still a concern among clinicians. For this reason, clinical prospective studies are strongly encouraged to determine the perioperative risks after oral surgical procedures and to define evidence-based guidelines for dental management of patients taking NOAs.

### Summary

Dabigatran etexilate, rivaroxaban, apixaban and edoxaban are four recently introduced drugs for the treatment of patients affected by various diseases and medical conditions that require use of extended-duration anticoagulant therapy. These new oral anticoagulant agents (NOAs) interfere with very specific steps of the coagulation cascade: dabigatran etexilate acts as a direct thrombin inhibitor (DTI), rivaroxaban, apixaban and edoxaban work as factor Xa inhibitors (FXaI). Since no specific reversal agents are available for these drugs (except for dabigatran etexilate), surgical dental treatment of patients taking NOAs requires particular attention in light of the bleeding risk associated to the procedure. Interruption of the NOA administration is generally not necessary for simple surgery as single dental extraction, while suspension has to be considered for extensive surgical treatments and/or in cases of patient’s comorbidities as renal impairment, advanced age or concomitant anti-platelet therapies.

Although the number of patients treated with NOAs is increasing, currently no established evidence-based guidelines are still available in literature for dental management of this cohort of patients. For this reason, further clinical prospective studies are needed to investigate the bleeding risk and haemostasis associated to surgical dental procedures.

## Abbreviations

NOA(s): novel oral anticoagulant(s)
LMWH: low-molecular-weight heparin
DTI: direct thrombin inhibitor
FXaI: factor Xa inhibitor
INR: international normalized ratio
TT: thrombin clotting time
ECT: ecarin clotting time
aPTT: activated partial thromboplastin time
DVT: deep venous thrombosis
NSAID: nonsteroidal antinflammatory drug
P-gp 1: P- glycoprotein 1
PT: prothrombin time
